# Diversity of Ascetosporeans Associated With Feather Hydroids in New Caledonia

**DOI:** 10.1111/jeu.70130

**Published:** 2026-10-06

**Authors:** Akinori Yabuki, Aya Yamazaki, Takehisa Yamakita, Tetsuya Miwa, Tatsuhiro Fukuba, Karine Olu, Takashi Toyofuku

**Affiliations:** ^1^ Institute for Extra‐Cutting‐Edge Science and Technology Avant‐Garde Research (X‐Star) Japan Agency for Marine‐Earth Science and Technology (JAMSTEC) Yokosuka Kanagawa Japan; ^2^ Advanced Institute for Marine Ecosystem Change (WPI‐AIMEC) Yokohama Kanagawa Japan; ^3^ Advanced Institute for Marine Ecosystem Change (WPI‐AIMEC) Sendai Miyagi Japan; ^4^ Research Institute for Global Change (RIGC) Japan Agency for Marine‐Earth Science and Technology (JAMSTEC) Yokosuka Kanagawa Japan; ^5^ Research Institute for Earth and Information Sciences (REIS) Japan Agency for Marine‐Earth Science and Technology (JAMSTEC) Yokohama Kanagawa Japan; ^6^ Research Institute for Marine Technology and Engineering (ENGD) Japan Agency for Marine‐Earth Science and Technology (JAMSTEC) Yokosuka Kanagawa Japan; ^7^ UMR6197, Biologie et Ecologie des Ecosystèmes Marins Profonds, CNRS, Ifremer Univ. Brest Plouzané France

## Abstract

Ascetosporea is a group of parasitic protists within Endomyxa that primarily infect marine invertebrates and can cause serious damage to fishery resources such as oysters and prawns. Although approximately 50 species have been formally described, environmental DNA (eDNA) studies suggest that their diversity remains largely underestimated, with many lineages yet to be discovered. Their ecological roles and host ranges are also poorly understood, partly because no complete life cycle has been elucidated, likely due to the difficulty of maintaining live cells in laboratory conditions. In this study, we investigated feather hydroids as potential hosts of ascetosporeans in New Caledonia, where this group has not been previously examined. Using metabarcoding with ascetosporean‐specific primers, we detected 46 operational taxonomic units (OTUs) from three hydroid individuals. Of these, 31 OTUs were affiliated with environmental clades ENDO‐3a and ENDO‐2, whereas two and four OTUs were assigned to Paradinida and Haplosporida, respectively. Notably, seven OTUs were consistently detected across all samples, suggesting that they may have been broadly associated with the feather hydroids in this environment. These findings indicate that ascetosporean diversity and host associations in marine ecosystems are substantially underestimated.

## 1 Introduction

Ascetosporea is a class‐level taxon within the phylum Endomyxa (Rhizaria) (Bass et al. 2019). Approximately 50 species have been formally described across five orders, and all are known to be parasites of invertebrates. Some members cause severe damage to marine resources, particularly in aquaculture, and have therefore received considerable attention in fisheries science (e.g., Utari et al. 2012; Carrasco et al. 2015). Furthermore, in the context of ongoing global climate change, there is concern that their impacts on host organisms and marine resources may intensify (Orenstein et al. 2022). Despite their ecological and economic importance, many aspects of ascetosporean biology remain poorly understood. Taxonomic studies have largely relied on infected host organisms, and stable cell cultures have only recently been reported (Yabuki et al. 2025). To date, the complete life cycle has not been resolved for any ascetosporean species. Environmental DNA (eDNA) studies further suggest that ascetosporean diversity is substantially underestimated, particularly in marine environments, where numerous environmental lineages remain undescribed (Ward et al. 2018; Berney et al. 2022). In addition, some ascetosporeans are suspected to utilize multiple host organisms during their life cycles (Carrasco et al. 2008), potentially including secondary hosts, implying that their ecological roles may be broader and more complex than currently recognized.

Benthic colonial hydrozoans that exhibit a pinnate branching structure are collectively called “feather hydroids” and are widely distributed marine organisms, including in deep‐sea environments (e.g., Prudkovsky et al. 2023; Fernandez and Marques 2018). They are often described as “animal forests” (Di Camillo et al. 2017) because they form structurally complex habitats that provide refuge and substrate for diverse marine organisms. As suspension feeders, feather hydroids may also contribute to carbon flux and play a role in the biological pump in marine ecosystems (Gili and Coma 1998). However, little is known about the parasites associated with feather hydroids. Although corals—another major group of habitat‐forming cnidarians—are known to host a variety of protistan associates and parasites (LaJeunesse et al. 2018; Siboni et al. 2010), feather hydroids have rarely been investigated from this perspective.

New Caledonia is an archipelago located in the Coral Sea in the southwestern Pacific Ocean, and its whole EEZ spanning 1.3 million km^2^ has been managed as a Marine Protected Area, the Coral Sea Natural Park, since 2014. However, its marine environments are increasingly threatened by global climate change (e.g., Taylor and Kumar 2016; Dutheil et al. 2021). Understanding the current state and dynamics of marine biodiversity in this region has therefore become an important research priority (Letourneur et al. 2023; Pelletier 2024). The region is also a hotspot of deep‐sea biodiversity (e.g., Kitahara and Cairns 2021), and seamounts have been identified as supporting high species richness (Samadi et al. 2006) and, particularly the shallowest ones, as biomass oases and potential refuges for highly mobile large predators also observed on coral reefs (Baletaud et al. 2023). Seamounts have been identified as key ecosystems in the Natural Park and focused for protection measures, with the creation in 2023 of the Norfolk ridge natural reserve. However, the regional deep‐sea biodiversity remains largely unexplored, and the high species richness and its variability between seamounts, observed since the first studies by Richer de Forges et al. (2000) is still poorly understood. To improve our understanding and predictive capacity of biodiversity distribution on seamounts off New Caledonia, a multidisciplinary observatory has been deployed since 2023 by the French Research Institute for Exploitation of the Sea (Ifremer; project ScInObs) in collaboration with JAMSTEC. A first mooring line was deployed in 2023 during the KASEAOPE‐1 cruise (Olu and Vic 2023) on the Stylaster seamount, part of the Norfolk Ridge. Mooring lines serve as key infrastructure for monitoring oceanographic processes and can support multiple observational devices (Lampitt et al. 2010). The mooring line off New Caledonia was equipped with several physical sensors and samplers including Autonomous Reef Monitoring Structures (ARMS), originally developed as standardized artificial substrates to sample cryptic benthic communities on coral reefs (Ransome et al. 2017), and biodiversity monitoring using ARMS has been ongoing. During the KASEAOPE‐2 cruise (Olu and Legrand 2024), ARMS were recovered and found to be heavily colonized by feather hydroids. In this study, we investigated feather hydroids as potential hosts of ascetosporeans using ascetosporean‐specific eDNA metabarcoding.

## 2 Materials and Methods

### 2.1 Deployment and Recovery of ARMS Panel System

The autonomous reef monitoring structures (ARMS) were composed of polyvinyl chloride panels connected by iron bolts. The ARMS were attached to the mooring line at a depth of 68 m from the top of the mooring line (Figure 1A). The mooring line, with a total length of 752 m, was deployed at the Station Stylaster seamount Northern Slope (Stn. SNS) (23°34.956′ S, 167°39.966′ E) on 5th May 2023 during the KASEAOPE‐1 cruise conducted with *R/V Antea* (Figure 1B), and recovered on 23rd October 2024 in the KASEAOPE‐2 cruise conducted with *R/V Antea*. The ARMS were subsequently detached from the line and then fixed in 99% ethanol at room temperature on board.

**FIGURE 1:**
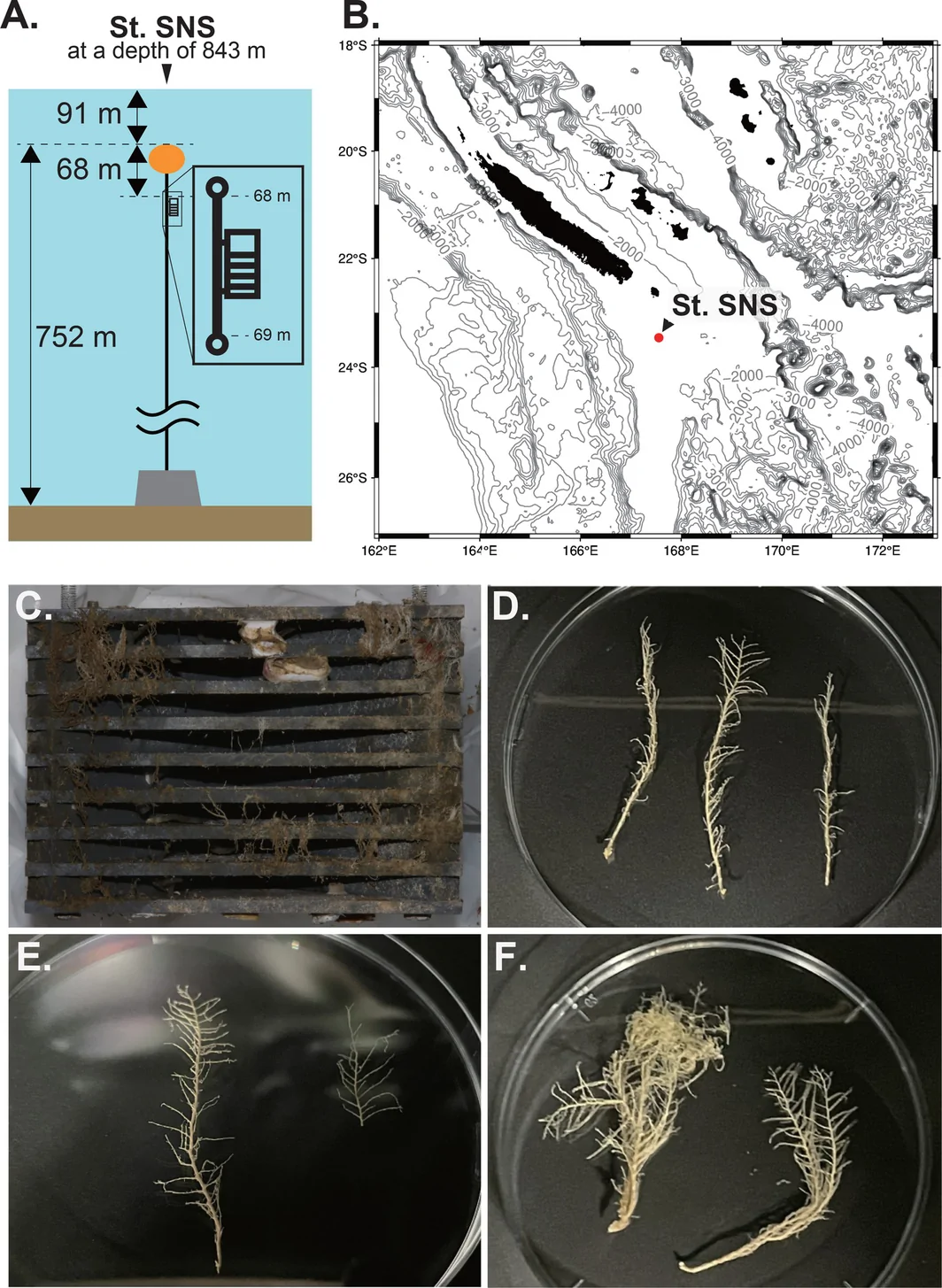
Summary of the research station and instruments. (A) Map of New Caledonia showing the location of Station STYLASTER Northern Slope (Stn. SNS). (B) Schematic illustration of the mooring system indicating the position of the ARMS. (C) Recovered ARMS with several feather hydroids growing on it. (D–F) Macrocolonia sp. #1, #2, and #3. The colonies were separated in a Petri dish but were originally connected as a single colony.

### 2.2 Collection of Feather Hydroids and DNA Extraction

The fixed ARMS were disassembled into individual panels after the cruise and each panel was kept in 99% ethanol. Numerous feather hydroids were attached to each panel (Figure 1C) and all appeared to belong to the same species. Three feather hydroid colonies on the top panel were randomly chosen and harvested from the panel (Figure 1D–F). The collected samples were kept in 99% ethanol until DNA extraction.

Total DNA from each colony was extracted from subsamples prepared by cutting the colonies into 2–8 cm pieces with a scalpel. DNA was extracted using the DNeasy Blood & Tissue Kit (Qiagen) according to the manufacturer's protocol.

### 2.3 PCR Amplification and Sequencing Analysis for Feather Hydroids

For species identification of feather hydroids, the 18S rRNA gene was amplified from each sample using the primer set 18SF and 18SR (Yabuki et al. 2010) and Ex Taq DNA polymerase (Takara) under the following conditions: an initial denaturation at 96°C for 1 min, followed by 30 cycles of denaturation at 94°C for 30 s, annealing at 55°C for 30 s, and extension at 72°C for 2 min, with a final hold at 4°C. The PCR products were subcloned using a Strataclone kit, and three clones from each sample were sequenced by Sanger sequencing. The obtained sequences were deposited in GenBank under the accession numbers LC917745–LC917753.

The obtained sequences were added to a newly prepared alignment file that included the major representative species from each family. The sequences were aligned by MAFFT v7.526 (Katoh et al. 2019) and the dataset for phylogenetic analyses was prepared with trimAl (Capella‐Gutiérrez et al. 2009) with ‐strict option. To ensure alignment quality, both the initial alignment and the trimmed dataset were visually inspected and manually curated. The maximum‐likelihood tree was reconstructed by IQ‐TREE 3 with the best‐fit model selected by ModelFinder option (Nguyen et al. 2015; Wong et al. 2026). Non‐parametric bootstrap values were calculated from 1000 replicates. Bayesian analysis was run using MrBayes v3.2.7a (Ronquist et al. 2012) with the GTR+Γ+I model. Markov chain Monte Carlo (one cold and three heated) with default chain temperatures was run for 1 × 10^7^ generations, with trees sampled every 1000 generations. The first 2.5 × 10^6^ generations were discarded as burn‐in because it was confirmed that the scores of the sampled log‐likelihood plateaued before 2.5 × 10^6^ generations.

### 2.4 PCR Amplification and Sequencing Analysis for Ascetosporeans

For understanding the ascetosporean diversity, the V5–V9 region of the 18S rRNA gene was amplified and sequenced by utilizing the primer sets reported in Ward et al. (2018). PCR amplification of three samples and subsequent sequencing analyses were performed by BitBiome (Tokyo, Japan). Amplification was performed using a nested PCR approach. V4fAsce and SB1n were used in the first round (40 cycles), followed by V5fAsce and EndoR1 in the second round (30 cycles). Both PCR rounds were carried out using KAPA HiFi HotStart Ready Mix. The amplification conditions were as follows: initial denaturation at 95°C for 5 min; 40 or 30 cycles of denaturation at 95°C for 30 s, annealing at 55°C for 1 min, and extension at 72°C for 1 min; followed by a final extension at 72°C for 10 min and holding at 4°C. Sequencing libraries were prepared using the SMRTbell prep kit 3.0 (PacBio) and sequenced on a PacBio Revio platform. The raw read data were deposited in GenBank under BioProject PRJDB40356 (PSUB047557).

Reads shorter than 600 bp or longer than 1000 bp were removed from the raw data because they were outside the target sequencing size range. The remaining sequences were analyzed using QIIME2 (qiime2‐amplicon‐2024.5) (Bolyen et al. 2019). Low‐quality reads were filtered out using the default settings, and ASVs were created using DADA2 (v2024.5.0) with the denoise‐single option (Callahan et al. 2016). OTU sequences were reconstructed with a 95% similarity threshold using VSEARCH v2.22.1 (Rognes et al. 2016). Minor sequences detected at < 0.05% abundance in each sample were removed, and the remaining sequences were subjected to BLASTn analysis. SILVA 136 was used as the database for BLASTn analysis, and sequences whose top hits corresponded to ascetosporeans were retained for subsequent analyses. The OTU sequences are provided upon request.

Sequences affiliated with ascetosporeans were aligned with those analyzed by Ward et al. (2018). In addition, several sequences recently reported to be affiliated with ascetosporeans were included in the alignment. Sequence alignment was performed using MAFFT with the ‐auto option (Katoh et al. 2019), and the dataset for phylogenetic analysis was prepared using trimAl with the ‐gappyout option (Capella‐Gutiérrez et al. 2009). OTUs containing gaps in more than half of their alignment positions were manually removed from the dataset. Phylogenetic analyses using maximum likelihood (ML) and Bayesian methods were conducted following the same procedures as those used for feather hydroids.

## 3 Results and Discussion

### 3.1 Molecular Identification of Feather Hydroids From New Caledonia

Nine cloned sequences obtained from three samples showed very high similarity to one another (99.50%–100%; Table S1) and formed a well‐supported clade (Figure 2). Slight sequence variation was detected within a single sample; however, clones from different samples, for example, clone #2 of sample No. 2 and clone #3 of sample No. 3, shared identical sequences (Table S1). The observed single nucleotide polymorphisms were located in both conserved and variable regions of 18S rRNA gene, and they may have been introduced due to PCR error. Hence, the three samples likely belong to the same species. In the phylogenetic analysis, the sequences clustered within the clade of Zygophylacidae, although statistical support was moderate (65% bootstrap support and 1.00 Bayesian posterior probability; Figure 2). We conducted additional phylogenetic analysis of the dataset that contained only core‐Macrocolonia sequences, but their positions were still not resolved with robust statistical support (Figure S1). Due to limited sequence availability, only three Zygophylacidae sequences were included in the analysis. Nevertheless, more than 70 species have been described in Zygophylacidae to date, many of which are distributed in deep‐sea environments (Gu et al. 2022). Several Zygophylacidae species have also been reported previously from the deep sea of New Caledonia. Therefore, the feather hydroid samples analyzed in this study may belong to the family Zygophylacidae. However, this assignment remains inconclusive based solely on phylogenetic evidence, and detailed morphological examination is required for definitive identification. Such morphological observations could not be conducted in the present study due to limitations in taxonomic expertise. In contrast, their placement within a subclade of Macrocolonia, labeled “core‐Macrocolonia,” was strongly supported (Figure 2). At minimum, the phylogenetic results clearly indicate that these samples belong to the order Macrocolonia (Figure 2B). Accordingly, we tentatively refer to them collectively as Macrocolonia sp. in this manuscript.

**FIGURE 2:**
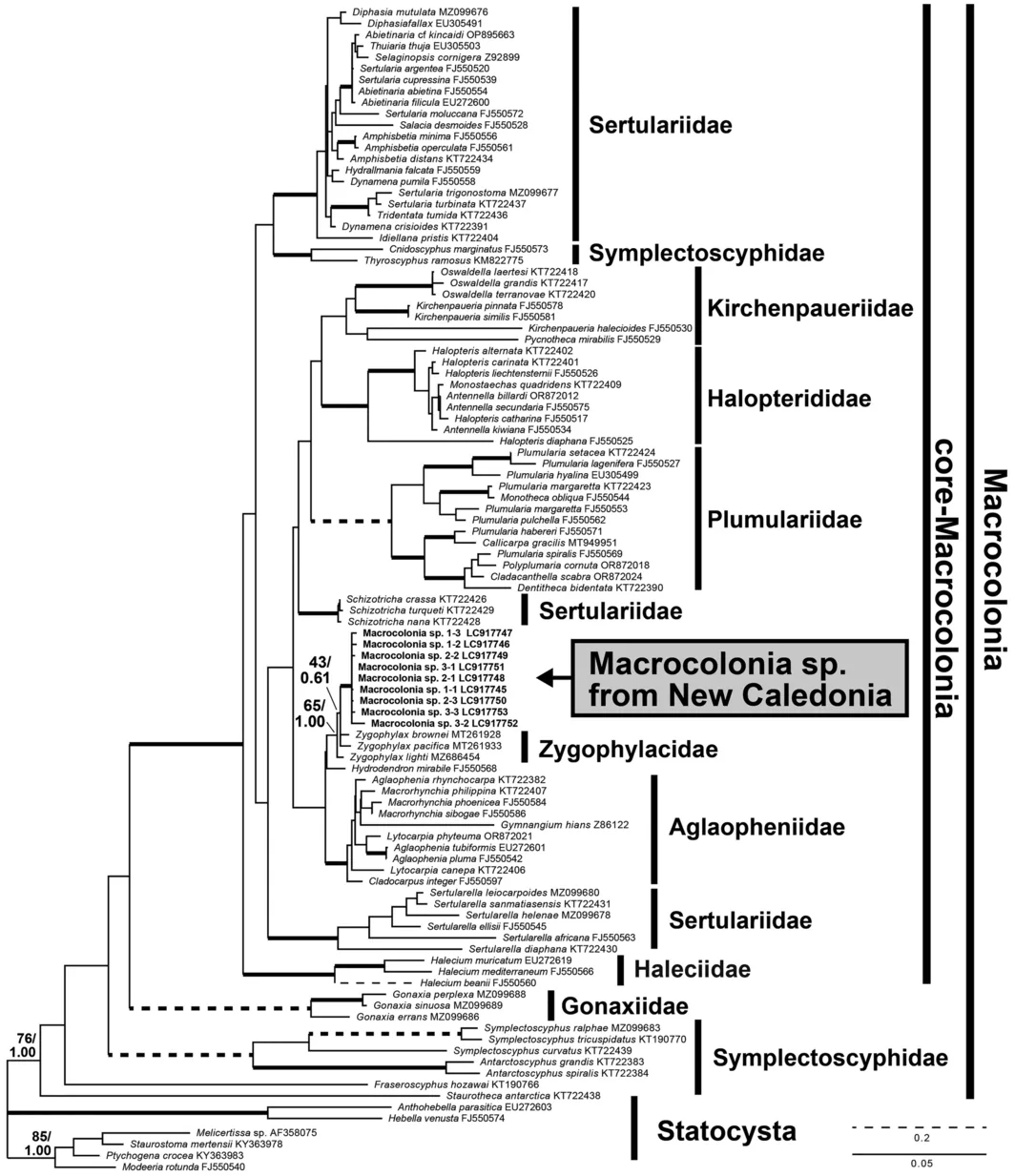
Phylogenetic tree of hydrozoan 18S rRNA gene sequences. The dataset comprised 103 OTUs and 1683 nucleotide positions, and was analyzed using maximum likelihood using the TN+F+R4 model. Branches supported by both > 90% ML bootstrap values and > 0.95 Bayesian posterior probabilities are indicated by bold lines.

### 3.2 Diversity of Ascetosporeans Affiliated With Macrocolonia sp. in New Caledonia

The phylogenetic analysis recovered the major clades reported in previous studies. Specifically, the monophyly of PARADIN‐1, Haplosporida, ENDO‐3b, SPP, and ENDO‐2 was individually recovered. In contrast, ENDO‐3a was not recovered as a monophyletic clade in our analyses. This result is not unexpected, as ENDO‐3a was also weakly supported in the study of Ward et al. (2018). The monophyly of the group termed “paradinids and early diverging Ascetosporea (PEDA)” by Ward et al. (2018) was likewise not well supported in our analysis, consistent with the findings of that previous study. Among the recovered clades, ENDO‐3b was strongly supported, whereas PARADIN‐1 and SPP received only weak support (Figure 3A). Although the monophyly of Haplosporida as a whole was not well supported, two of its subclades—Haplo‐B and core‐Haplosporida (core‐H)—were each strongly supported (Figure 3A). These results suggest that the circumscription of Haplosporida requires careful reconsideration, particularly regarding which clades should be included alongside core‐Haplosporida. Furthermore, taxonomic identification of members of Haplo‐B and other environmental clades currently assigned to Haplosporida is strongly needed to clarify their evolutionary relationships and stabilize the classification of the group.

**FIGURE 3:**
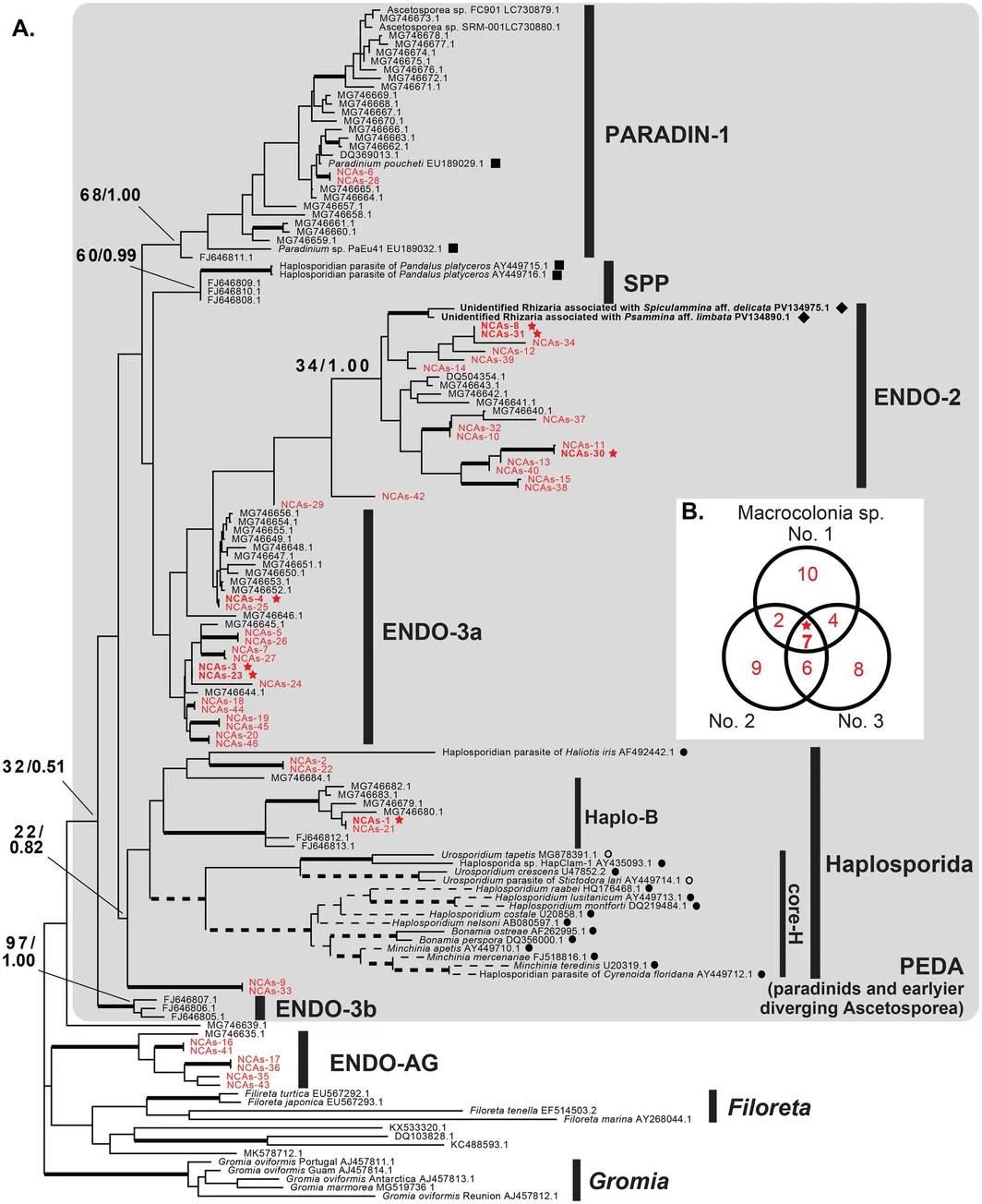
Results of sequence analyses of New Caledonia Ascetosporean sequences (NCAs). (A) Phylogenetic tree of endomyxean 18S rRNA gene sequences. The dataset comprised 140 OTUs and 750 nucleotide positions, and was analyzed using maximum likelihood under the TN+F+R4 model. Branches supported by both > 90% ML bootstrap values and > 0.95 Bayesian posterior probabilities are indicated by bold lines. Support values for several branches discussed in the manuscript are additionally shown. OTUs detected in the present study are shown in red, whereas environmental sequences are indicated by accession numbers only. OTUs consistently detected in all three samples are marked with red stars. Sequences with identified organismal origins are indicated by a black square (crustacean), a black diamond (xenophyophore), a black circle (mollusk), and an open circle (flatworm parasitizing a mollusk). (B) Venn diagram showing the number of detected ascetosporean sequences from three Macrocolonia samples.

In total, 46 distinct ascetosporean‐affiliated sequences (NCAs; New Caledonia Ascetosporea sequences) were detected from three samples of Macrocolonia sp., and seven sequences were consistently detected across all samples (Figure 3B). We consider the sequencing depth for each sample to be sufficient to capture the major lineages, as rarefaction curves reached a plateau in all cases (Figure S2). This interpretation is further supported by the observation that a comparable number of ascetosporean sequences was recovered from Macrocolonia sp. No. 3, despite the fact that approximately four times more raw reads were obtained from this sample than from the other two due to resource limitations (Table 1).

**TABLE 1:** Summary of sequencing and bioinformatic processing results.

	No. of CCS reads	No. of CCS reads (600–1000 bp)	No. of filtered reads	No. of denoised ASVs	No. of non‐chimeric ASVs	No. of OTUs (95%)	No. of OTUs (> 0.05%)	No. of NCAs sequences
Macrocolonia sp. No. 1	418,913	238,106	224,545	215,486	60,336	51	41	23
Macrocolonia sp. No. 2	337,459	145,387	139,471	126,456	36,543	47	39	24
Macrocolonia sp. No. 3	1,626,858	1,364,799	1,287,550	338,132	338,132	104	43	25

The PARADIN‐1 clade is considered to correspond to the order Paradinida. In our analysis, two sequences (NCAs‐6 and NCAs‐28) branched within a subclade of PARADIN‐1 with high statistical support. Currently, the order includes only a single genus, *Paradinium*, comprising three described species, all of which are known parasites of copepods (Shields 1994). Although the complete life cycle of *Paradinium* spp. has not been fully elucidated, previous observations suggest that zoospores hatched from the gonosphere—an aggregation of cyst cells—directly infect another copepod host (Shields 1994; Skovgaard and Daugbjerg 2008). Given this established host association, the placement of NCAs‐6 and NCAs‐28 within PARADIN‐1 raises the possibility that these sequences represent lineages with life strategies distinct from those of described *Paradinium* species. Accordingly, the host range of PARADIN‐1 members may be broader than currently recognized. Considering the phylogenetic diversity within PARADIN‐1, such an expansion of host range would not be unexpected. For example, within core‐Haplosporida, documented hosts include mollusks (Arzul and Carnegie 2015), annelids (Caullery and Mesnil 1905), and crustaceans (Davies et al. 2020). This broad host spectrum among related ascetosporean lineages suggests that host specificity within PARADIN‐1 may likewise be more diverse than presently assumed. Efforts to better understand the diversity of PARADIN‐1 have recently advanced through the establishment of cultures (Yabuki et al. 2025). Further exploration targeting a wider range of potential host organisms will likely contribute substantially to clarifying the ecological and evolutionary diversity of this clade.

Four sequences, NCAs‐1, ‐2, ‐21, and ‐22, branched within Haplosporida sensu lato, and the affiliation of NCAs‐1 and NCAs‐21 with Haplo‐B was strongly supported (Figure 3A). The Haplo‐B clade has previously consisted exclusively of environmental sequences derived from deep‐sea samples (Ward et al. 2018), and the organismal origin of these sequences has remained unclear. Therefore, this represents the first report of Haplo‐B sequences from non‐deep‐sea samples. This finding may be explained if their principal host organisms are feather hydroids, which are abundant in deep‐sea environments. Because NCAs‐1 was consistently detected in all three samples, it is likely predominantly associated with Macrocolonia sp. in New Caledonia. Its taxonomic identity may be clarified through future investigations focusing on Macrocolonia sp. NCAs‐2 and NCAs‐22 formed a clade and branched with the haplosporidian parasite of *Haliotis iris* , although this relationship was not statistically supported (Figure 3A). They may represent an independent lineage within Haplosporida and/or within PEDA as a whole, and clarification of their organismal identity is anticipated to improve our understanding of ascetosporean diversity.

While the monophyly of ENDO‐3a was not recovered, 15 NCAs were affiliated with either of the sequences previously assigned as ENDO‐3a member (Figure 3A). No potential host organism of ENDO‐3a members has been suggested previously, therefore, this information provides a clue to their identification and some members are at least associated with Macrocolonia sp. Since three sequences, that is, NCAs‐3, ‐4, and ‐23, were consistently detected from all samples, they may also be predominantly associated with Macrocolonia sp. in New Caledonia. This is also the case for ENDO‐2 because 15 sequences were detected in total and three sequences were consistently detected from all three samples. Two ENDO‐2 sequences were recently reported from a metagenomic analysis of xenophyophores (Gastineau et al. 2025), but it remains unclear how they live in association with xenophyophores. It can also be considered that the host organisms of ENDO‐2 are also diverse as is the case of the other clades.

Within the PEDA clade, four sequences, NCAs‐9, ‐29, ‐33, and ‐42, were not affiliated with any previously recognized clades (Figure 3A). Their phylogenetic positions were not fully resolved; however, NCAs‐9 and NCAs‐33 formed a clade with high statistical support. Nevertheless, given the overall low support values, it remains uncertain whether these sequences should be regarded as members of PEDA. In addition, six NCAs formed a clade with an environmental sequence (MG746635.1) that was not included in PEDA in the previous study. We collectively designate this group as the ENDO‐AG clade (Figure 3A). The name ENDO‐AG reflects their possible affiliation with Ascetosporea (A) and Gromiidea (G; *Gromia* and *Filoreta*). Clarifying the organismal identity of these lineages will also be important for understanding the diversity and early evolution of Ascetosporea. Targeted analyses of feather hydroids, including Macrocolonia sp., may represent one of the most feasible approaches for achieving this goal.

### 3.3 Ascetosporean Diversity and Ecological Significance

Feather hydroids are widely distributed worldwide, including in deep‐sea environments; however, their potential role as host organisms for ascetosporean parasites has not previously been investigated. It was unexpected that 46 distinct sequences were detected from Macrocolonia sp., and that these sequences were phylogenetically diverse within Ascetosporea. Although the possibility cannot be excluded that some of these sequences originated from parasites infecting small organisms attached to Macrocolonia sp., they may nonetheless be regarded, from a broader perspective, as being associated with Macrocolonia sp. Another possibility is that some of these sequences originated from environmental particles or prey items trapped by the hydroids during feeding. In contrast, NCAs‐3, 8, 23, and 31 were consistently detected from three samples with high relative abundance (Table S2), suggesting that, at least, they may have a stronger association with Macrocolonia sp. than other detected ascetosporeans. Since feather hydroids have never been considered as host organisms for ascetosporeans, it is important to investigate whether other hydrozoans in other oceans also represent accessible host organisms for ascetosporeans or not. It has been increasingly shown over the last two decades that apicomplexans and their relatives are associated with corals (e.g., Janouškovec et al. 2013), hence, it is therefore not surprising that hydrozoans may also be broadly associated with ascetosporeans. Other marine animals are also important research targets as the possible ascetosporean hosts, and further studies on these groups are still needed.

It is also of considerable interest to determine how diverse ascetosporeans, including non‐identified lineages, are cytologically associated with host organisms. It is well known that parasitic strategies differ among ascetosporean lineages; for example, *Haplosporidium* spp., *Bonamia* spp., and *Paradinium* spp. are histozoic (intra‐tissue), cytozoic (intracellular), and coelozoic (within body cavities) parasites, respectively (Shields 1994; Hine 2020). It can also be considered that some members may be commensals or symbionts of host animals. Studies on the function of microeukaryotes associated with animal hosts have received increasing attention recently (del Campo et al. 2020). The parasitic style of ascetosporeans detected from New Caledonia may also be diverse, and their ecological impacts on the host are possibly different among lineages. In addition, it could also be considered that some ascetosporeans have a free‐living or amphizoic lifestyle. Although all known ascetosporeans are parasites of invertebrates, some members may have trophic flexibility, like that reported from diplonemids (Prokopchuk et al. 2022). Such information is key to understanding their ecological roles in the oceans.

The sequencing approach employed in the present study was unable to detect several ascetosporean lineages, such as Mikrocytida and Paramyxida, because their 18S rRNA gene sequences are too divergent to be amplified by the primer set used here. These groups are also ecologically important research targets (Abbott and Meyer 2014; Hartikainen et al. 2014; Carrasco et al. 2015; Ward et al. 2016), and dedicated studies focusing on them are strongly warranted. Future studies using other barcoding genes are also expected in this context. Ascetosporeans have long been regarded as one of the most poorly understood groups of protists. However, knowledge of their diversity has steadily accumulated, and culturing methods for some lineages have recently been established (Yabuki et al. 2025). Although many aspects of their evolution and ecological roles still remain unresolved, significant progress is anticipated in the coming decade.

## Author Contributions

Takehisa Yamakita (T.Y.), Tatsuhiko Fukuba (T.F.), and Takashi Toyofuku (T.T.) designed and prepared the ARMS for the pilot line; Karine Olu (K.O.) was the chief scientist of the Kaseaope‐1 and 2 cruises, and T.Y. and Tetsuya Miwa (T.M.) also joined the research cruise to deploy and recover the ARMS; Akinori Yabuki (A.Y.), T.Y., T.M., T.F., K.O., and T.T. handled the recovered ARMS and subdivided the samples. A.Y. and Aya Yamazaki analyzed the samples and the data. A.Y. wrote and all authors edited the manuscript.

## Funding

The ScInObs “Science, Innovation et Observatoires sous‐marins” project is fully funded by Ifremer under the exceptional scientific investment programme (PIE Ifremer). The cost of the vessel is supported by the French Oceanographic Fleet.

## Conflicts of Interest

The authors declare no conflicts of interest.

## Supporting information


**Figure S1:** Phylogenetic tree of core‐Macrocolonia 18S rRNA gene sequences. The dataset comprised 69 OTUs and 1682 nucleotide positions, which was prepared from the original alignment by Trimal with “‐gappyout” option. The tree was reconstructed by IQ‐TREE 3 with the best‐fit model: TN+F+I+R3 model. Non‐parametric ML bootstrap values > 80% were only shown and the branches supported by > 0.95 Bayesian posterior probabilities shown by bold lines; the values for the branches around Macrocolonia spp. were exceptionally shown.
**Figure S2:** The relationship between sequencing depth and the number of the reconstructed OTUs. Note that this number correspond to that shown in “# of OTUs (95%)” in Table 1.
**Table S1:** Sequence similarity among 18S rRNA gene sequences of Macrocolonia sp.
**Table S2:** Top 10 OTUs by relative abundance in each Macrocolonia sample.

## Data Availability

The sequence data analyzed in the present study were deposited to Genbank under the accession numbers LC917745–LC917753 and BioProject: PRJDB40356 (PSUB047557).
