# Supplementary material for: Diversity of Ascetosporeans Associated With Feather Hydroids in New Caledonia
Source: J Eukaryot Microbiol. 2026 Oct 6;73(6):e70130. doi: 10.1111/jeu.70130 (PMC13639817; doi:10.1111/jeu.70130)
Supplement: Supplementary file 1 [file JEU-73-e70130-s001.docx]

**Supplementary Information**

Table S1

Sequence similarity among 18S rRNA gene sequences of Macrocolonia sp.

|  | Macrocolonia sp.  No. 1 #1 | Macrocolonia sp.  No. 1 #2 | Macrocolonia sp.  No. 1 #3 | Macrocolonia sp.  No. 2 #1 | Macrocolonia sp.  No. 2 #2 | Macrocolonia sp.  No. 2 #3 | Macrocolonia sp.  No. 3 #1 | Macrocolonia sp.  No. 3 #2 | Macrocolonia sp.  No. 3 #3 |
| --- | --- | --- | --- | --- | --- | --- | --- | --- | --- |
| Macrocolonia sp. No. 1 #1  (LC917745) | NA | 99.94 | 99.94 | 99.94 | 99.72 | 99.89 | 99.89 | 99.50 | 99.94 |
| Macrocolonia sp. No. 1 #2  (LC917746) |  |  | 99.89 | 99.89 | 99.67 | 99.83 | 99.83 | 99.45 | 99.89 |
| Macrocolonia sp. No. 1 #3  (LC917747) |  |  |  | 99.89 | 99.67 | 99.83 | 99.83 | 99.45 | 99.89 |
| Macrocolonia sp. No. 2 #1  (LC917748) |  |  |  |  | 99.78 | 99.94 | 99.94 | 99.56 | 100.00 |
| Macrocolonia sp. No. 2 #2  (LC917749) |  |  |  |  |  | 99.72 | 99.72 | 99.34 | 99.78 |
| Macrocolonia sp. No. 2 #3  (LC917750) |  |  |  |  |  |  | 99.89 | 99.50 | 99.94 |
| Macrocolonia sp. No. 3 #1  (LC917751) |  |  |  |  |  |  |  | 99.50 | 99.94 |
| Macrocolonia sp. No. 3 #2  (LC917752) |  |  |  |  |  |  |  |  | 99.56 |
| Macrocolonia sp. No. 3 #3  (LC917753) |  |  |  |  |  |  |  |  | NA |

Table S2

Top 10 OTUs by relative abundance in each Macrocolonia sample.

|  | Macrocolonia sp. No.1 | | Macrocolonia sp. No.2 | | Macrocolonia sp. No.3 | |
| --- | --- | --- | --- | --- | --- | --- |
|  | OTU# | Relative Abundance | OTU# | Relative Abundance | OTU# | Relative Abundance |
| 1 | NCAs-31 | 0.256712411 | NCAs-23 | 0.16161782 | NCAs-31 | 0.315098837 |
| 2 | NCAs-8 | 0.209427208 | NCAs-32 | 0.108037107 | NCAs-8 | 0.254684561 |
| 3 | NCAs-23 | 0.152976664 | NCAs-10 | 0.10505432 | NCAs-33 | 0.126811423 |
| 4 | NCAs-3 | 0.118121851 | NCAs-3 | 0.104698574 | NCAs-9 | 0.086197106 |
| 5 | NCAs-25 | 0.013391673 | NCAs-31 | 0.090249843 | NCAs-23 | 0.061499651 |
| 6 | NCAs-4 | 0.012098913 | NCAs-8 | 0.073447719 | NCAs-3 | 0.036311263 |
| 7 | NCAs-28 | 0.006646115 | NCAs-40 | 0.049886435 | NCAs-32 | 0.008162493 |
| 8 | NCAs-6 | 0.005767701 | NCAs-44 | 0.046137427 | NCAs-10 | 0.006423527 |
| 9 | NCAs-21 | 0.005568815 | NCAs-13 | 0.039925567 | NCAs-44 | 0.002868702 |
| 10 | NCAs-29 | 0.005353355 | NCAs-33 | 0.03094984 | NCAs-21 | 0.002300877 |

The OTUs detected in all three samples are highlighted with a gray background. Relative abundance was calculated as follows:

Relative abundance = Number of raw reads assigned to each OTU / Total number of raw reads, including non-ascetosporean sequences.

Figure S1

Phylogenetic tree of core-Macrocolonia 18S rRNA gene sequences. The dataset comprised 69 OTUs and 1,682 nucleotide positions, which was prepared from the original alignment by Trimal with ‘-gappyout’ option. The tree was reconstructed by IQ-TREE 3 with the best-fit model: TN+F+I+R3 model. Non-parametric ML bootstrap values >80% were only shown and the branches supported by >0.95 Bayesian posterior probabilities shown by bold lines; the values for the branches around Macrocolonia spp. were exceptionally shown.

Figure S2

The relationship between sequencing depth and the number of the reconstructed OTUs. Note that this number correspond to that shown in ‘# of OTUs (95%)’ in Table 1.
